# Positive association between fibrinogen-to-albumin ratio and preeclampsia risk in pregnant women of advanced maternal age: implications for clinical risk assessment

**DOI:** 10.3389/fcvm.2025.1638353

**Published:** 2025-10-31

**Authors:** Nuoni Wang, Shihao Liu, Xiaoqin Duan, Hui Xie, Liangqing Ge, Sulan Huang

**Affiliations:** ^1^ Department of Electrophysiology, Changde Hospital, Xiangya School of Medicine, Central South University (The First People’s Hospital of Changde City), Changde, China; ^2^ Department of Cardiology, Changde Hospital, Xiangya School of Medicine, Central South University (The First People’s Hospital of Changde City), Changde, China

**Keywords:** fibrinogen-to-albumin ratio, preeclampsia, advanced maternal age, inflammatory biomarker, pregnancy risk, early screening

## Abstract

**Introduction:**

Preeclampsia is a serious hypertensive disorder unique to pregnancy, with increased prevalence among women of advanced maternal age (AMA). Given the central role of inflammation and vascular dysfunction in the development of preeclampsia, this study aimed to evaluate the relationship between the fibrinogen-to-albumin ratio (FAR) multiplied by 100 (FAR × 100) and risk of preeclampsia in this high-risk group.

**Methods:**

A retrospective cohort of 2,296 pregnant women of advanced maternal age (≥35 years) was analyzed. The exposure variable, FAR × 100, was calculated as fibrinogen (g/L) divided by albumin (g/L) and multiplied by 100. PE was diagnosed based on the established clinical criteria. Participants were grouped into tertiles according to FAR × 100 levels. Both crude and adjusted logistic regression models were used to assess associations. Nonlinear relationships were assessed using restricted cubic spline (RCS) analysis, and subgroup analyses were conducted based on maternal characteristics.

**Results:**

The average age was 37.8 ± 2.7 years, with a preeclampsia prevalence of 14.3%. A higher FAR × 100 score was significantly associated with increased odds of developing preeclampsia. In the fully adjusted model, each unit increase in FAR × 100 raised the likelihood of preeclampsia by 8% (odds ratio [OR] = 1.08; 95% confidence interval [CI]: 1.06–1.10; *p* < 0.001). Compared to the lowest tertile, participants in the highest tertile had a significantly elevated risk (OR = 1.56; 95% CI: 1.11–2.19). The positive association between FAR × 100 and preeclampsia remained consistent across the subgroups, including those stratified by body mass index (BMI), red cell distribution width (RDW), gravidity, and family history of hypertension, with no evidence of a significant interaction.

**Conclusion:**

FAR × 100 is independently associated with preeclampsia among women of AMA and may serve as a low-cost practical biomarker for early clinical risk stratification in prenatal care.

## 1 Introduction

Preeclampsia is a hypertensive disorder unique to pregnancy that remains a global primary contributor to maternal and neonatal complications and death (1, 2). Preeclampsia is clinically identified by the emergence of elevated blood pressure (≥140/90 mmHg) and proteinuria (≥300 mg over 24 h) after 20 weeks of gestation in women with previously normal blood pressure (2). Affecting approximately 2%–8% of pregnancies worldwide, preeclampsia is frequently associated with severe adverse outcomes, such as premature delivery, fetal growth restriction, placental abruption, and an increased risk of long-term cardiovascular disease in affected mothers (2–4). Although substantial progress has been made toward understanding this condition, its precise pathogenesis remains elusive. Current hypotheses focus on disrupted placental development, widespread endothelial cell injury, and excessive maternal inflammatory responses as the major driving mechanisms (2, 5). These systemic alterations are often mirrored in maternal blood through measurable biomarkers such as inflammatory mediators and coagulation-related factors, which may hold promise for early identification and risk assessment (3, 5).

The prevalence of advanced maternal age (AMA), defined as pregnancy at or beyond the age of 35 years, has been rising globally, particularly in high-income countries, due to delayed childbearing influenced by social and reproductive factors (6). This demographic shift has been accompanied by an increase in age-associated obstetric complications, among which preeclampsia remains one of the most serious threats to maternal and neonatal health (7, 8). Women with AMA are more likely to develop metabolic and cardiovascular disturbances, including diabetes, chronic hypertension, and obesity, which are strongly associated with preeclampsia (9). These comorbidities contribute to a cascade of pathophysiological processes such as endothelial dysfunction, angiogenic imbalance, and oxidative stress, all of which play central roles in the onset and progression of preeclampsia (8, 9). These risk-enhancing factors underscore the importance of identifying effective and affordable biomarkers to support early clinical risk assessments in this vulnerable population.

The fibrinogen-to-albumin ratio (FAR) is an inflammatory marker predictive of adverse outcomes in vascular diseases (10). Since preeclampsia involves inflammatory activation and coagulative dysfunction, FAR may represent an integrated biomarker for these pathophysiological processes (11–13). Fibrinogen, a protein with pro-inflammatory properties, tends to increase during vascular injury and systemic stress (14). Conversely, albumin is a negative acute-phase protein associated with endothelial stability, which typically declines under inflammatory conditions (15). Consequently, the FAR offers a consolidated metric that mirrors the interplay between systemic inflammation and vascular function. The prognostic utility of FAR has been elucidated in cardiovascular disorders, such as coronary artery disease (16), diabetes-related cognitive impairment (17), cerebral small vessel disease (18),chronic heart failure (19), ischemic stroke (10), and hypertension-related vascular risk (14). Moreover, elevated FAR levels have been observed in pregnancy-related conditions, including gestational diabetes mellitus (20) and recurrent miscarriages (21). Despite this growing body of evidence, the predictive relevance of FAR for preeclampsia, particularly among women of AMA, remains to be thoroughly explored. Given the predisposition of this population to endothelial dysfunction and chronic low-grade inflammation, the FAR may serve as a cost-effective and accessible indicator to facilitate early risk assessment in clinical settings.

We, therefore, aimed to investigate the relationship between FAR × 100 and the risk of preeclampsia in pregnant women of AMA. The objectives were two-fold: 1) to assess the predictive validity of FAR × 100 for preeclampsia risk in this high-risk population, and 2) to explore the potential clinical utility of FAR × 100 as a tool for early risk stratification and intervention in AMA pregnancies.

## 2 Materials and methods

### 2.1 Study population

This retrospective cohort investigation enrolled 2,296 pregnancies with advanced maternal age (≥35 years), spanning data collection from January 2013 through December 2023 at Changde Hospital, China. Participants underwent tertile stratification according to fibrinogen-to-albumin ratio (FAR) to examine associations with preeclampsia development. The biomarker index was computed by dividing fibrinogen concentrations by albumin concentrations, with analysis groups established as follows (14):

FAR=fibrinogen(g/L)/albumin(g/L)

**Figure 1:**
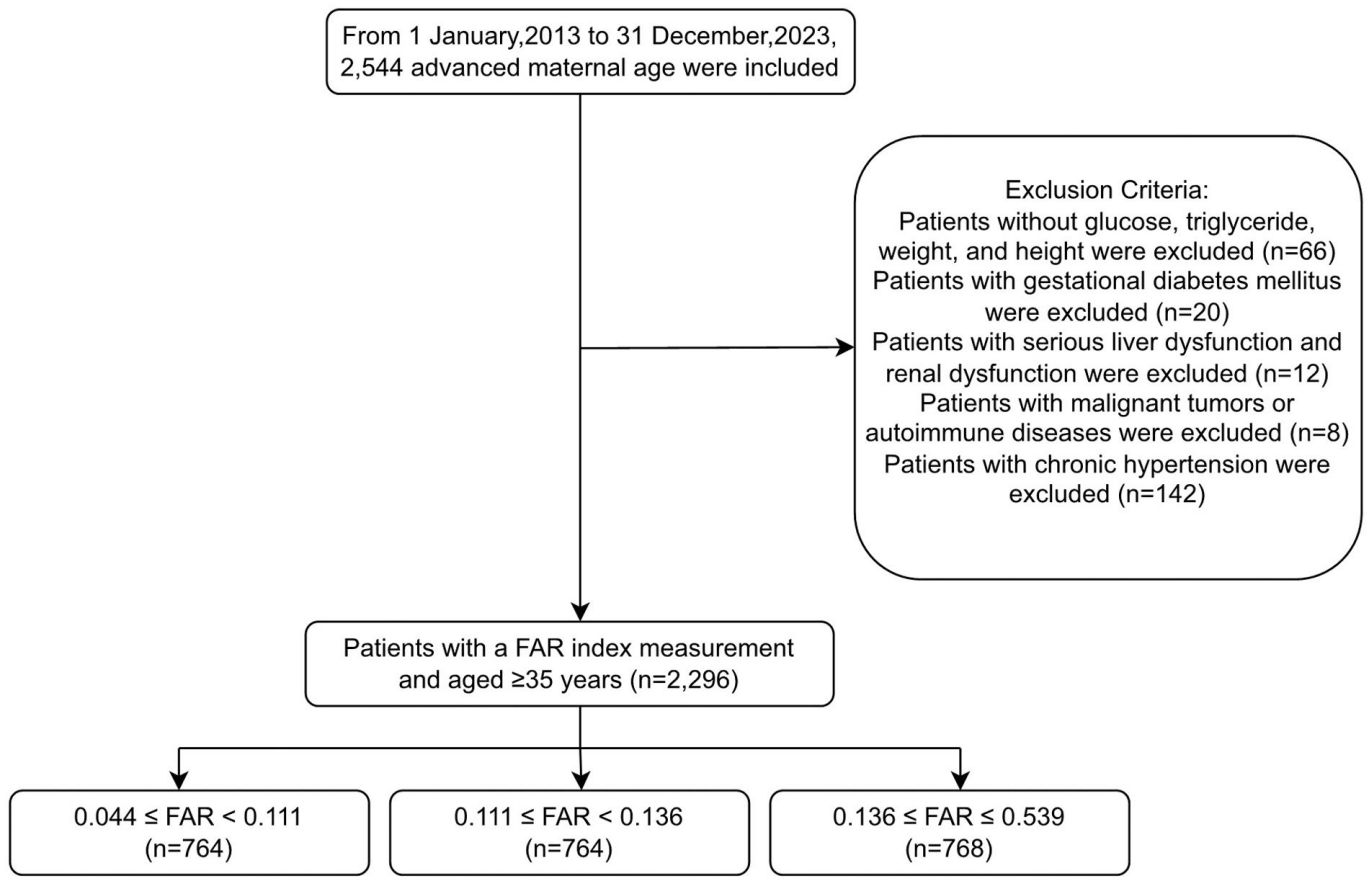
Flowchart of participant selection and enrollment process for the study.

The study initially included 2,544 pregnant women of AMA (≥35 years) who met the inclusion criteria of a single pregnancy, available medical records, and complete laboratory tests. Women were excluded if they had missing glucose, triglyceride (TG), weight, or height measurements (*n* = 66); gestational diabetes mellitus (*n* = 20); severe liver or renal dysfunction (*n* = 12), which could significantly affect fibrinogen and albumin levels; malignant tumors or autoimmune diseases (*n* = 8); or chronic hypertension (*n* = 142). After exclusion, 2,296 women remained, who were further categorized into groups based on FAR: Tertile 1, FAR < 0.111; Tertile 2, 0.111≤ FAR < 0.136; and Tertile 3, FAR ≥ 0.136. Among these, 328 women had preeclampsia and 1,968 did not (Figure 1). Patients with severe liver or renal dysfunction were excluded to avoid confounding effects on fibrinogen and albumin levels, which could compromise the validity of FAR × 100 as a predictive biomarker.

### 2.2 Laboratory analysis

Maternal data, including age, body mass index (BMI), glucose, TG, and family history of hypertension, were extracted from the hospital records. Blood samples for fibrinogen and albumin analysis were obtained during routine early pregnancy prenatal visits, typically occurring at 11–12 weeks of gestation. The interval between blood collection for FAR analysis and preeclampsia diagnosis (confirmed after 20 weeks of gestation according to established criteria) varied among participants, supporting the predictive rather than diagnostic nature of our biomarker assessment. Blood pressure measurements (systolic and diastolic) were performed at multiple time points in accordance with the American Heart Association guidelines (22). Laboratory analyses, including maternal uric acid (UA) and albumin (ALB) levels, were conducted using automated equipment (AU5800-A, Beckman Coulter, Tokyo, Japan).

### 2.3 Statistical analysis

Statistical analyses were performed using R software (http://www.R-project.org, The R Foundation) and Free Statistics software version 2.1.1. Continuous variables are summarized as mean ± standard deviation (SD) or median (interquartile range, IQR) and compared across groups using one-way analysis of variance or the Kruskal–Wallis test, depending on the data distribution. Categorical variables are presented as frequencies and percentages and were compared using the chi-squared test.

Multivariate logistic regression analyses were performed to assess the association between FAR × 100 and risk of preeclampsia. Three models were constructed: Model 1 was unadjusted; Model 2 was adjusted for age, gravidity, and parity; and Model 3 was adjusted for all variables in Model 2 plus family history of hypertension, creatinine (CR), body mass index (BMI), and glucose (GLU). Odds ratios (ORs) with 95% confidence intervals (CIs) are reported. FAR × 100 was analyzed both as a continuous variable and as a tertile, and a trend test was conducted.

Nonlinear associations between FAR × 100 and preeclampsia were explored using restricted cubic spline (RCS) regression, adjusting for the same covariates as those in Model 3. Subgroup analyses were conducted to examine potential effect modifications by age, gravidity, and family history of hypertension, and *P*-values for interactions were calculated to assess the statistical heterogeneity across the strata. A two-tailed *p*-value < 0.05 was considered statistically significant.

## 3 Results

### 3.1 Study population and clinical characteristics

Table 1 provides detailed baseline characteristics across the three FAR tertiles. Significant differences were found in both the demographic and clinical variables. As FAR increased, participants in the highest tertile (Tertile 3) showed significantly higher BMI, white blood cell (WBC) count, TG levels, and fibrinogen, and lower albumin levels than those in Tertile 1 (all *p* < 0.001). Additionally, the prevalence of hypertensive disorders of pregnancy (HDP) was more pronounced in the higher FAR tertiles, with 16.2% and 16.7% of participants in Tertiles 2 and 3, respectively, having HDP, compared to 9.9% in Tertile 1 (*p* < 0.001). Furthermore, participants with a higher FAR also had higher age, gravidity, platelet count, red cell distribution width (RDW), and several biochemical markers, such as alanine aminotransferase (ALT), UA, and lipid profiles, with significant differences between the groups (*p* < 0.05). Notably, no significant differences were observed in serum creatinine, aspartate aminotransferase (AST), or urine protein levels across the FAR tertiles. These findings suggest a potential association between higher FAR levels and increased risk factors for preeclampsia, such as elevated BMI and WBC, triglyceride, and fibrinogen levels.

**Table 1:** Baseline characteristics according to fibrinogen-to-albumin ratio.

FAR tertiles	Total (*N* = 2,296)	Tertile 1 (*N* = 764)	Tertile 2 (*N* = 764)	Tertile 3 (*N* = 768)	*P*
BMI	27.6 ± 3.4	26.9 ± 3.6	27.7 ± 3.2	28.1 ± 3.4	<0.001
GLU	5.1 ± 1.5	5.1 ± 1.1	5.0 ± 1.0	5.3 ± 2.2	<0.001
TG	4.0 ± 1.5	3.9 ± 1.3	4.0 ± 1.1	4.1 ± 1.8	0.005
HDP					<0.001
No	1,968 (85.7)	688 (90.1)	640 (83.8)	640 (83.3)	
Yes	328 (14.3)	76 (9.9)	124 (16.2)	128 (16.7)	
AGE	37.8 ± 2.7	37.4 ± 2.5	37.8 ± 2.4	38.1 ± 3.0	<0.001
GRAVIDITY	3.7 ± 1.8	3.6 ± 1.6	3.7 ± 1.7	3.9 ± 2.1	<0.001
RDW	14.6 ± 3.5	14.8 ± 4.5	14.4 ± 1.4	14.5 ± 3.7	0.029
WBC	9.1 ± 2.6	8.7 ± 2.3	8.9 ± 2.4	9.7 ± 2.9	<0.001
RBC	3.9 ± 1.8	4.1 ± 3.1	3.8 ± 0.4	3.7 ± 0.5	<0.001
HB	114.8 ± 13.8	114.2 ± 14.0	116.4 ± 13.9	113.9 ± 13.4	<0.001
HCT	0.3 (0.3, 0.4)	0.4 (0.3, 0.4)	0.4 (0.3, 0.4)	0.3 (0.3, 0.4)	0.003
PLT	188.1 ± 57.6	179.8 ± 52.4	188.6 ± 64.2	195.9 ± 54.4	<0.001
ALB	35.9 ± 4.5	38.6 ± 5.3	35.2 ± 3.2	34.0 ± 3.4	<0.001
GLOBULIN	27.8 ± 4.1	27.1 ± 3.5	27.9 ± 3.6	28.6 ± 4.8	<0.001
ALT	13.5 (10.0, 21.0)	12.0 (10.0, 20.0)	14.0 (10.0, 19.0)	14.0 (10.0, 25.5)	<0.001
AST	20.0 (17.0, 26.0)	19.0 (17.0, 25.0)	20.0 (16.0, 26.0)	20.0 (16.0, 27.2)	0.357
BUN	3.6 ± 2.0	3.7 ± 3.2	3.5 ± 1.0	3.5 ± 1.0	0.017
CR	46.8 ± 9.9	46.9 ± 10.5	46.4 ± 9.2	47.3 ± 9.8	0.241
TC	6.1 ± 0.9	6.1 ± 0.7	6.2 ± 0.9	6.1 ± 0.9	<0.001
HDL	1.8 ± 0.3	1.8 ± 0.2	1.9 ± 0.5	1.8 ± 0.2	<0.001
LDL	3.1 ± 0.5	3.0 ± 0.5	3.1 ± 0.5	3.0 ± 0.6	<0.001
D-Dimer	1.9 (1.2, 2.7)	1.7 (1.0, 2.6)	1.9 (1.4, 2.6)	2.0 (1.3, 3.1)	<0.001
FIB	4.5 ± 1.4	3.5 ± 0.6	4.4 ± 0.5	5.7 ± 1.8	<0.001
UA	311.2 ± 84.5	297.9 ± 89.4	317.0 ± 78.0	318.8 ± 84.4	<0.001
HR	91.7 ± 11.6	90.6 ± 11.5	91.2 ± 10.3	93.2 ± 12.8	<0.001
Family history of hypertension					<0.001
No	1,672 (72.8)	656 (85.9)	528 (69.1)	488 (63.5)	
Yes	624 (27.2)	108 (14.1)	236 (30.9)	280 (36.5)	
Urine protein					0.437
No	1,256 (54.7)	432 (56.5)	408 (53.4)	416 (54.2)	
Yes	1,040 (45.3)	332 (43.5)	356 (46.6)	352 (45.8)	

Data are shown as mean ± SD, median (IQR), or *n* (%). BMI, body mass index; GLU, glucose; TG, triglycerides; RDW, red cell distribution width; WBC, white blood cells; RBC, red blood cells; HB, hemoglobin; HCT, hematocrit; PLT, platelets; ALB, albumin; GLOBULIN, globulin; ALT, alanine aminotransferase; AST, aspartate aminotransferase; BUN, blood urea nitrogen; CR, creatinine; TC, total cholesterol; HDL, high-density lipoprotein; LDL, low-density lipoprotein; D-dimer, fibrin degradation product; UA, uric acid.

### 3.2 Univariate and multivariate logistic analysis results

Table 2 presents the results of the multivariate logistic regression analysis. In the unadjusted model, a significant positive association was observed between the FAR × 100 and risk of preeclampsia, with an OR of 1.09 (95% CI: 1.06–1.11, *p* < 0.001). After adjusting for age, gravidity, and parity in Model 1, the association remained significant (OR = 1.09, 95% CI: 1.06–1.11, *p* < 0.001). In Model 2, further adjustments for family history of hypertension, creatinine, BMI, and glucose maintained the association, though the OR slightly decreased to 1.08 (95% CI: 1.06–1.10, *p* < 0.001). Analysis by FAR × 100 tertiles showed that participants in the second and third tertiles had significantly higher odds of developing preeclampsia compared to the first tertile (OR for Tertile 2 = 1.90, 95% CI: 1.36–2.66, *p* < 0.001; OR for Tertile 3 = 1.56, 95% CI: 1.11–2.19, *p* = 0.011 in Model 2). A significant trend was observed across the FAR × 100 tertiles, with *P*-values for the trends remaining significant in the unadjusted and adjusted models (*p* < 0.001 for the unadjusted model and Model 1, *p* = 0.023 for Model 2) (Table 2).

**Table 2:** Results of multivariate logistic regression conducted to evaluate the relationship between the fibrinogen-to-albumin ratio and risk of preeclampsia.

FAR × 100	Non-adjusted model		Adjust I model		Adjust II model	
	OR (95% CI)	*P*	OR (95% CI)	*P*	OR (95% CI)	*P*
Continuous	1.09 (1.06, 1.11)	<0.001	1.09 (1.06, 1.11)	<0.001	1.08 (1.06, 1.10)	<0.001
FAR × 100						
Tertile 1	1.0		1.0		1.0	
Tertile 2	1.75 (1.29, 2.38)	<0.001	1.79 (1.32, 2.44)	<0.001	1.90 (1.36, 2.66)	<0.001
Tertile 3	1.81 (1.34, 2.45)	<0.001	1.87 (1.38, 2.55)	<0.001	1.56 (1.11, 2.19)	0.011
P for trend		<0.001		<0.001		0.023

Unadjusted model: no covariates were included.

Model I adjustment: controlled for age, gravidity, and parity.

Model II adjustment: further included family history of hypertension, creatinine (CR), body mass index (BMI), and glucose (GLU).

CI, confidence interval; OR, odds ratio.

### 3.3 RCS curve analysis results

**Figure 2:**
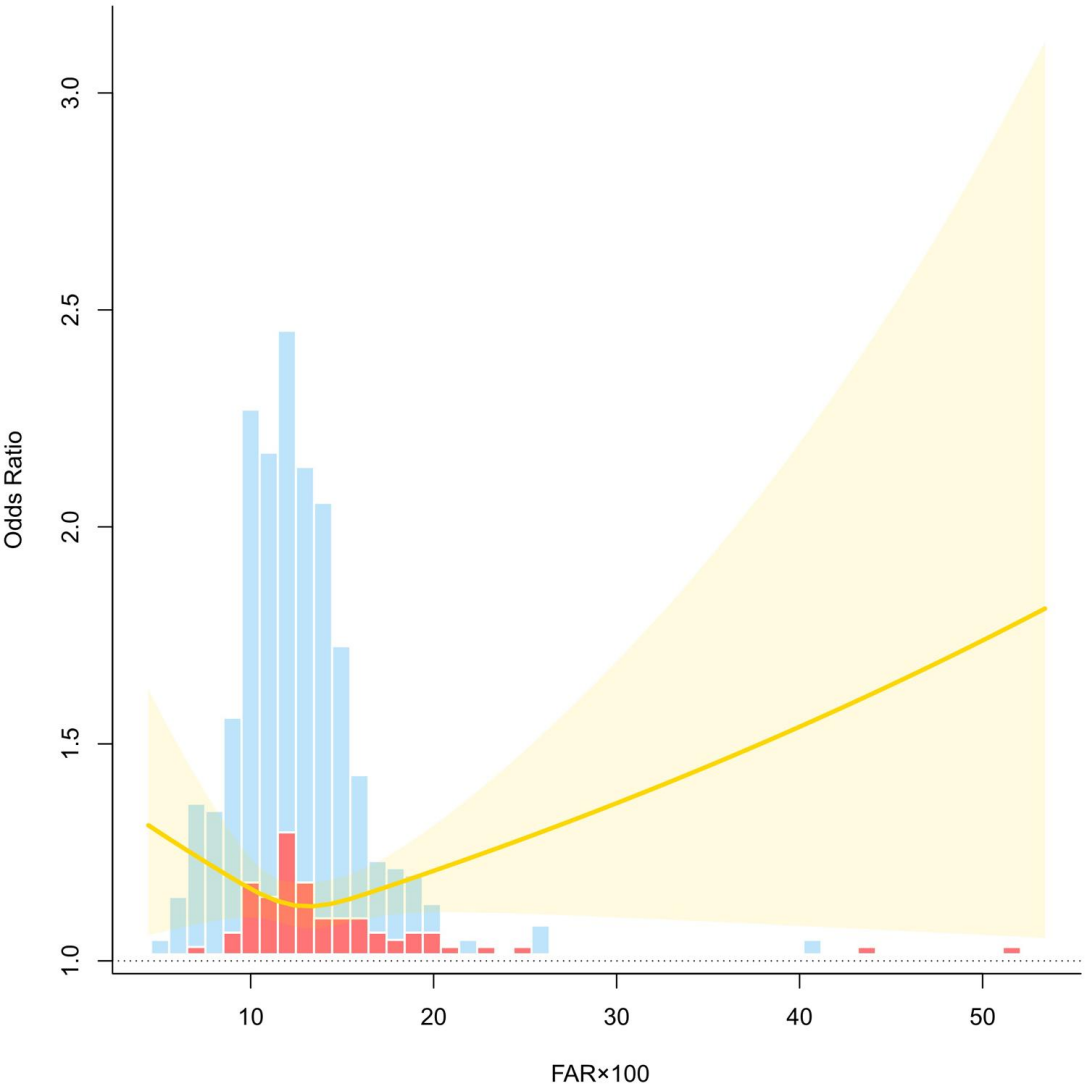
RCS curve analysis.

An RCS model was fitted to explore the nonlinear association between FAR × 100 and risk of preeclampsia. After adjusting for age, gravidity, parity, family history of hypertension, creatinine levels, BMI, and glucose levels, the curve revealed a J-shaped relationship. At lower levels of FAR × 100, the OR remained relatively stable and close to 1.0, suggesting no significant increase in risk. However, as FAR × 100 exceeded approximately 15, the OR increased rapidly with a widening CI, indicating growing uncertainty at higher FAR values. The upward trajectory of the curve at higher FAR levels suggests a dose-response trend, where an elevated FAR × 100 is associated with an increased preeclampsia risk. The shaded region denotes the 95% confidence band, and the histogram bars represent the distribution of cases and controls across the FAR × 100 range. These findings support the nonlinear effect of FAR on preeclampsia risk and underscore the potential threshold beyond which FAR becomes a clinically relevant marker of elevated risk (Figure 2).

### 3.4 Subgroup forest plot

Subgroup analyses were conducted to explore whether the association between FAR × 100 and preeclampsia risk varied across the clinical strata. Adjusted ORs and 95% CIs were calculated using multivariate logistic regression models by treating FAR × 100 as a continuous variable per unit increase. Interaction terms were evaluated with significance set at *P* < 0.05.

**Figure 3:**
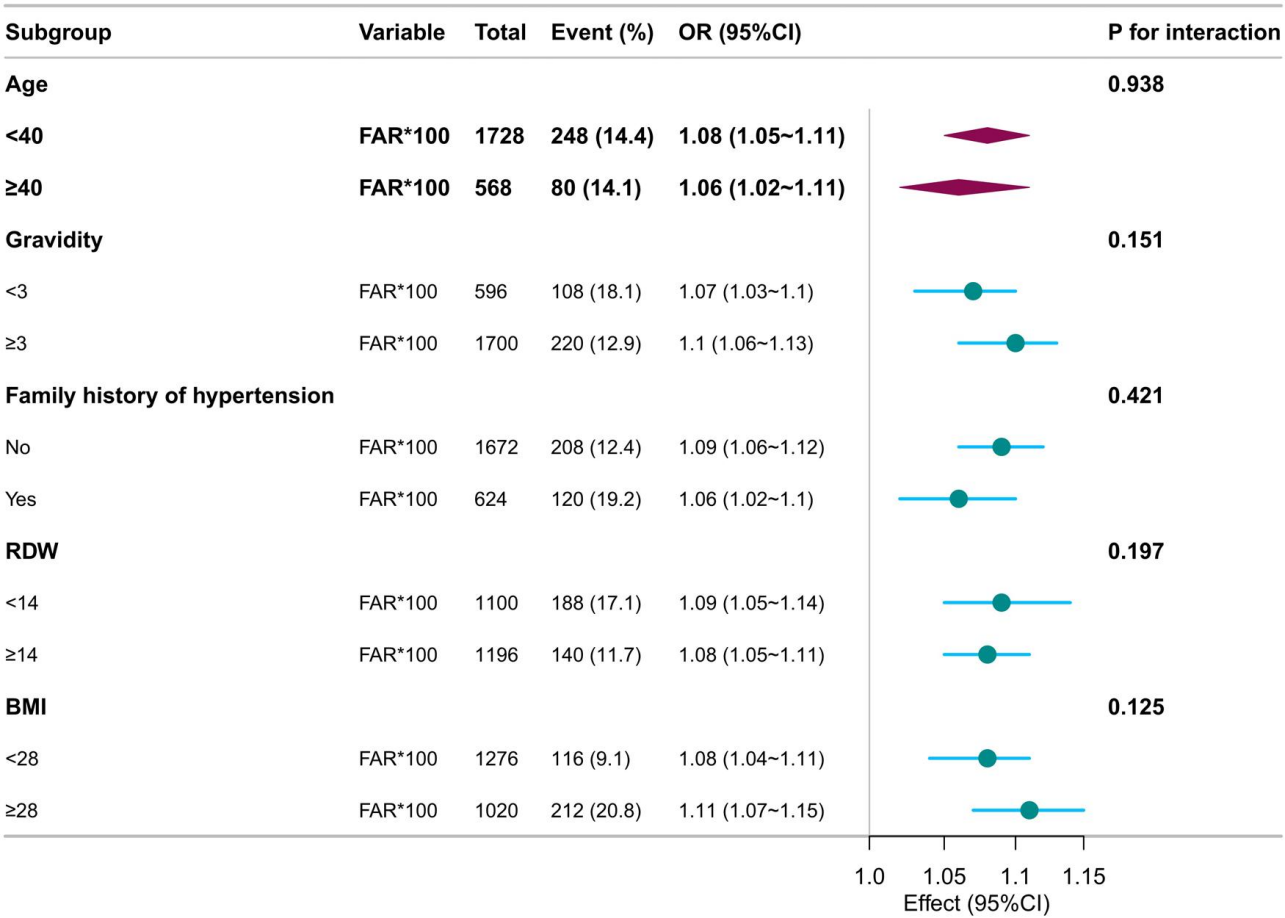
Subgroup forest plot.

The positive association between FAR × 100 and preeclampsia persisted across all subgroups. Among participants with a family history of hypertension, the OR was 1.06 (95% CI: 1.02–1.10), compared to 1.09 (95% CI: 1.06–1.12) in those without, with no statistically significant interaction (*P* = 0.421). Stronger associations were observed in women with gravidity ≥3 (OR: 1.10, 95% CI: 1.06–1.13), BMI ≥28 kg/m^2^ (OR: 1.11, 95% CI: 1.07–1.15), and RDW ≥14% (OR: 1.08, 95% CI: 1.05–1.11). In all analyses, the interaction *P*-values were above 0.05, suggesting no significant effect modifications. Overall, FAR × 100 demonstrated a consistent predictive relationship with preeclampsia risk across various demographic and metabolic conditions, supporting its utility as a robust early risk indicator (Figure 3).

## 4 Discussion

Our results confirm a robust and independent association between FAR × 100 and preeclampsia risk in women with AMA. A dose-response trend was observed, with higher FAR × 100 levels corresponding to an increased incidence of preeclampsia. This relationship remained consistent across both continuous and categorical analyses, underscoring FAR × 100 as a reliable marker in this high-risk population, even after adjusting for key covariates, including age, gravidity, parity, BMI, fasting glucose, serum creatinine, and family history of hypertension.

Subgroup analyses further demonstrated that the association between FAR × 100 and risk of preeclampsia remained stable across various clinical profiles. Elevated FAR × 100 was significantly linked to preeclampsia among women aged <40 years, those with a family history of hypertension, BMI ≥28 kg/m^2^, and RDW <14%. All interaction *P*-values exceeded 0.05, suggesting no significant heterogeneity across the subgroups. These findings reinforce FAR × 100 as a universally applicable and independent predictor, supporting its integration into early screening strategies for preeclampsia risk stratification in AMA.

The FAR × 100 index incorporates two clinically relevant biomarkers, fibrinogen and albumin, which reflect the systemic inflammatory and vascular states. Fibrinogen, a positive acute-phase reactant, plays a central role in coagulation and immune activation. Albumin, a negative acute-phase protein, is involved in endothelial health and nutritional status. An elevated FAR × 100 typically signals a shift toward pro-inflammatory and prothrombotic conditions, both of which are closely associated with the onset of preeclampsia (23–25).

The observed association between increased FAR × 100 and preeclampsia risk could be explained by multiple interconnected mechanisms. First, endothelial dysfunction, a hallmark of preeclampsia, is aggravated by reduced NO bioavailability, heightened vascular tone, and increased permeability (23, 26). Elevated fibrinogen and reduced albumin levels may contribute to these pathological changes through their effects on inflammation and vascular function (13, 23). Second, oxidative stress resulting from placental ischemia and reperfusion injury accelerates trophoblast damage and the release of anti-angiogenic factors, further impairing vascular function (26–28). Third, chronic subclinical inflammation, more prevalent in older pregnant women, stimulates pro-inflammatory cytokine release (e.g., TNF-α, IL-6), which exacerbates endothelial injury and vascular maladaptation (23, 27, 28).

AMA is associated with metabolic derangements such as insulin resistance, arterial stiffness, and increased baseline vascular inflammation, all of which contribute to heightened vulnerability to preeclampsia (2, 25). Consistent with our stratified analysis, the FAR × 100-preeclampsia association remained significant in women with and without traditional risk factors, such as obesity, family history of hypertension, or multiparity, indicating that its predictive value may not be limited to specific subgroups. Therefore, FAR × 100 may serve as a practical and integrative biomarker, capturing both inflammatory and vascular alterations. Its utility in the early identification of high-risk individuals can facilitate timely intervention and improve maternal outcomes. The exclusion of patients with moderate or severe preeclampsia after 20 weeks of gestation, as well as those with severe hepatic and renal dysfunction at enrollment, ensured the validity of our biomarker measurements while allowing for a focused assessment of predictive performance primarily in those with mild preeclampsia.

Our findings align with previous research demonstrating the roles of fibrinogen and albumin in preeclampsia pathophysiology (4, 29). Our study further highlights the predictive value of the FAR, particularly in women of advanced maternal age. Elevated fibrinogen levels are associated with systemic inflammation, hypercoagulability, and endothelial activation—central mechanisms in the development of HDP (14, 16). In contrast, hypoalbuminemia indicates endothelial glycocalyx deterioration with impaired barrier integrity, leading to enhanced vascular permeability and capillary extravasation (30). Such endothelial dysfunction, combined with maternal vascular underperfusion (31), contributes to placental insufficiency and restricted fetal growth (2).

In contrast to studies evaluating individual biomarkers, our analysis of the composite FAR × 100 index provides enhanced risk stratification. Recent cardiovascular studies demonstrate FAR's superior predictive capacity for clinical outcomes compared with fibrinogen or albumin alone, by capturing the balance between inflammatory and protective pathways (29, 32, 33). Moreover, studies in obstetric populations have shown that FAR increases significantly in patients with severe preeclampsia and may serve as a potential marker of disease severity and progression (29, 34).

Given that preeclampsia is characterized by vascular dysfunction,oxidative stress, and chronic low-grade inflammation (4, 13), and AMA increases the risk of preeclampsia, FAR × 100 may serve as a particularly valuable predictive tool in this high-risk population. Our subgroup analyses further supported this finding, showing that FAR × 100 maintained a significant association with preeclampsia in women with AMA as well as across strata of BMI, gravidity, RDW, and family history of hypertension, with no evidence of interaction across subgroups (all *P* > 0.05). Therefore, our results extend previous findings by confirming that FAR × 100 is not only independently associated with preeclampsia but is also consistently predictive across diverse clinical profiles and may serve as a feasible, accessible, and cost-effective biomarker for the early identification of women at high risk for preeclampsia.

Our subgroup analysis sheds light on the heterogeneity of the association between FAR × 100 and preeclampsia risk across maternal characteristics. Although the association remained significant in both age groups, the effect size was marginally higher in women under 40 years (OR: 1.08, 95% CI: 1.05–1.11) than in those aged 40 years and older (OR: 1.06, 95% CI: 1.02–1.11), suggesting that FAR × 100 may serve as a more broadly applicable predictor rather than one restricted to AMA. This increased susceptibility arises from heightened oxidative stress and impaired endothelial function (35, 36). Pregnancy complications are also linked to increased placental dysfunction and delayed trophoblast remodeling, leading to compromised uteroplacental perfusion and adverse pregnancy outcomes (37, 38).

Beyond age, the association between FAR × 100 and preeclampsia risk remained robust across other key maternal characteristics including BMI, RDW, gravidity, and family history of hypertension. No significant interactions were observed, indicating consistent predictive performance across these subgroups. FAR × 100 demonstrated consistent predictive value across different gravidity groups, indicating that reproductive history does not significantly modulate maternal vascular responses. Particularly, nulliparous individuals are more vulnerable to endothelial injury and exaggerated inflammatory responses during their first exposure to trophoblast invasion. These results are in line with recent evidence showing that FAR reflects both the pro-inflammatory status (via fibrinogen) and diminished vascular reserve (via albumin), thus serving as a holistic indicator of maternal vascular stress (16, 29).

Clinically, our findings support the inclusion of FAR × 100 in prenatal risk stratification models. Given its ease of measurement, cost-effectiveness, and accessibility across clinical settings, FAR × 100 may provide an alternative or adjunct to the more complex biomarker panels currently in use. Previous studies have demonstrated FAR's value in assessing cardiovascular risk (16), while research has also highlighted the significant cardiovascular impact of hypertensive disorders in pregnancy (39). The consistent association across subgroups enhances its clinical utility, suggesting that FAR × 100 may aid in identifying at-risk individuals even in the absence of traditional risk markers, and integrating FAR × 100 into early prenatal screening could identify high-risk patients who may benefit from prophylactic interventions such as low-dose aspirin or intensified monitoring (2, 3).

Low-dose aspirin effectively reduces the incidence of preeclampsia in high-risk populations, particularly when initiated before 16 weeks of gestation (4). Early recognition through FAR × 100 could facilitate timely preventive strategies and improve maternal-fetal outcomes (11). This is especially critical in AMA populations where standard risk indicators may not fully capture the underlying vascular pathology (40). Our study adds to the growing body of literature advocating for a composite biomarker that integrates the inflammatory and vascular dimensions of pregnancy-related conditions to guide personalized obstetric care.

Despite its strengths, this study had some limitations. First, the retrospective design limited the ability to establish causal relationships, and although we adjusted for a range of potential confounders, a residual bias may still be present. Second, the study was conducted in a single-center setting, which may have affected the generalizability of the findings. Additionally, the cross-sectional nature of biomarker measurements limits the ability to assess temporal changes in the FAR and their direct impact on the development of preeclampsia over the course of pregnancy. Third, although subgroup analysis revealed consistent associations between FAR × 100 and preeclampsia across clinical variables such as maternal age, gravidity, BMI, RDW, and family history of hypertension, the absence of a statistically significant interaction did not preclude potential effect modification in larger or more diverse cohorts. Fourth, our study did not compare the predictive performance of FAR × 100 with established multi-marker models or imaging-based screening tools, thus limiting its current positioning within the broader spectrum of prenatal risk assessment strategies. Fifth, considering the specific timing of sample collection, our study lacked a sensitivity analysis stratified by exact gestational weeks of blood collection, which limited our ability to strengthen causal inferences regarding the predictive utility of FAR × 100. Moreover, we did not include patients who developed moderate or severe preeclampsia after 20 weeks of gestation, focusing primarily on those with mild preeclampsia. This exclusion further represents a limitation of our study.

Future studies should aim to prospectively validate the FAR × 100 index in diverse populations, particularly in multicenter settings, to assess its external validity. Longitudinal studies with repeated measurements of FAR during pregnancy would help elucidate the temporal relationship between FAR changes and the development of preeclampsia. Such studies should also incorporate sensitivity analyses stratified by precise gestational timing of biomarker collection to strengthen causal inference. Additionally, research on the biological mechanisms underlying the association between FAR × 100 and preeclampsia, such as specific inflammatory markers, endothelial dysfunction, and oxidative stress pathways, will provide a deeper understanding of how this biomarker functions in predicting pregnancy complications. Comparative modeling studies are also warranted to evaluate whether FAR × 100 adds incremental predictive value beyond existing risk models or whether it may serve as a simpler proxy when access to advanced diagnostics is limited. Our study suggests that FAR × 100 is a promising biomarker in the identification of pregnant women at a high risk of preeclampsia, particularly in AMA cohorts. Its simplicity, cost-effectiveness, and potential for early intervention make it an attractive addition to current prenatal screening strategies. Given the consistent predictive value of FAR × 100 across maternal subgroups in our analysis, future prospective studies should investigate its utility in real-world prenatal care settings and explore whether FAR-guided strategies can inform targeted interventions to reduce the burden of preeclampsia.

## 5 Conclusion

This study identified a significant and independent link between elevated FAR × 100 and an increased risk of preeclampsia among pregnant women of AMA. The association remained stable in the multivariable-adjusted and nonlinear analyses. Subgroup analysis confirmed the predictive value of FAR × 100 across various maternal profiles, with relatively stronger associations observed in younger women, those with higher gravidity, and those without a family history of hypertension.

## Data Availability

The original contributions presented in the study are included in the article/Supplementary Material, further inquiries can be directed to the corresponding author.
